# Can Medical Interventions Serve as ‘Criminal Rehabilitation’?

**DOI:** 10.1007/s12152-016-9264-9

**Published:** 2016-06-27

**Authors:** Gulzaar Barn

**Affiliations:** University of Oxford, Oxford, UK

**Keywords:** Moral bioenhancement, Rehabilitation, Punishment

## Abstract

‘Moral bioenhancement’ refers to the use of pharmaceuticals and other direct brain interventions to enhance ‘moral’ traits such as ‘empathy,’ and alter any ‘morally problematic’ dispositions, such as ‘aggression.’ This is believed to result in improved moral responses. In a recent paper, Tom Douglas considers whether medical interventions of this sort could be “provided as part of the criminal justice system’s response to the commission of crime, and for the purposes of facilitating rehabilitation (Douglas in *Journal of Ethics* 18(2): 101–122, 2014).” He suggests that they could “at least in some cases, permissibly be provided without valid consent (Douglas in *Journal of Ethics* 18(2): 101–122, 2014)” as a form of rehabilitative punishment. He argues for this conclusion by ‘parity of reasoning,’ starting from the currently accepted practice of non-consensual incarceration. His argument appears to be dependent on the successful defence of the following two claims: (1) that non-consensual incarceration is a morally justifiable practice, and (2) that there is no meaningful distinction between the forcible imposition of this practice, and the forcible imposition of medical interventions on prisoners. From both claims, he deduces (3): if non-consensual incarceration is morally justifiable, so is the non-consensual imposition of medical correctives, in some cases. In this paper, I begin by suggesting that the basic argument behind the Parity Claim (2) results in a *reductio ad absurdum*, whereby any practice that is sufficiently similar to incarceration in the ways Douglas presents, would also be considered permissible. This appears to be an unpalatable conclusion, casting doubt on the soundness of a key premise. Douglas appears to offer no means of deciding which practices are sufficiently similar to incarceration in terms of harm and threat to agency, and of the practices that I will present, which do seem to be, it does not seem that he could rule them out in any principled way. Next I turn to dispute claim (1) relating to the purported justifiability of incarceration on rehabilitative grounds. If successful, this attack causes a break in reasoning from the justifiability of incarceration, to the justifiability of medical interventions (2). Medical interventions would then require an alternative, independent justification, through outlining the ways in which they are conducive to a particular aim of punishment, without relying on the justifiability of incarceration. This argument has not been provided, and I suggest that attempts to do so may run into difficulty. I untangle and make explicit the various assumptions made in Douglas’ contention that medical interventions “might be thought conducive to rehabilitation in some offenders,” locating my critique in the wider debate on the causes of crime. Finally, I seek to challenge the normative weight of the Parity Claim (2), arguing that to show that two practices are comparable in some sense is not sufficient to show that both are equally as permissible (3). Other social purposes must be considered, leading me to suggest that the forcible imposition medical correctives falls beyond the appropriate remit of the criminal justice system.

## Preliminaries

It is important to note that Douglas is clear to distance himself from our current practice of incarceration, which is criticised for its overcrowded conditions and the high risks of rape and assault that it imposes upon prisoners. It is not this *actual* incarceration that he takes to be justifiable. Rather, he assumes that it would be justifiable to impose a hypothetical minimal incarceration model, which would be safer, and provide real opportunities for political participation, legal representation and education. Douglas does not make any attempt to defend, or provide an independent argument for the claim that such a model of incarceration would be justifiable. Instead, the motivation for assuming its justifiability is that such a model would be “widely (though not universally) accepted,”^1^ and so is worth taking seriously for the sake of argument.

### The Consent Requirement

Douglas focuses on the theoretical question as to whether it would be permissible to impose medical interventions on prisoners without their valid consent. He is motivated by the belief that current debates surrounding the use of medical correctives on prisoners rest on a mistaken assumption about consent, which he refers to as the Consent Requirement. Arguments made by opponents of medical interventions in the criminal context tend to be premised upon this requirement, which holds that “medical correctives can only permissibly be provided with the valid consent of the offender who will undergo the intervention.”^2^ Such an assumption, Douglas argues, seems to follow naturally from the parallel, undisputed claim in medical ethics, that therapeutic medical interventions should not, except in certain special circumstances, be provided to a competent adult patient without consent.

When opponents of criminal medical interventions argue that such a practice would be coercive, and so any consent obtained would be invalid, they are implicitly invoking the Consent Requirement as a premise in their argument against its permissibility. This debate has been particularly played out in regards to the offer of chemical castration to prisoners, in exchange for early release. This is said to be coercive, as prisoners already lack a fundamental sense of liberty and ‘ability to choose,’ and so any such decision to undertake a medical intervention would not be freely and voluntarily chosen, thus rendering their consent invalid. Douglas seeks to sidestep this critique, laying out an alternative line of response: that the Consent Requirement may not be as defensible as such theorists require.

He argues this through the development of what I will critique as the ‘Parity Claim’, and analogises the use of medical correctives to the practice of incarceration. Foundational to the analogy is the claim that:

“it is widely thought that the state may permissibly do things to criminal offenders without their consent that it could not permissibly do to others without (and in some cases even with) consent. Thus, for example, it would ordinarily be grossly wrong to incarcerate someone without consent, but in the context of criminal justice, non-consensual incarceration is widely thought to be permissible.”^3^

Douglas continues that our current practice of non-consensual incarceration, or minimal incarceration, places serious constraints on individuals’ freedom of movement and freedom of association. He argues that of the several differences one could appeal to between minimal incarceration and medical correctives, in order to justify why the consent requirement should hold for criminal medical correctives despite being overridden for incarceration, none are successful. Crucially, he asks, if the goal of rehabilitation is sufficiently important to justify the imposition of non-consensual incarceration, how could this same goal fail to justify the non-consensual imposition of at least some medical correctives?

### The Parity Claim

Douglas argues that the Consent Requirement runs into difficulty when applied to the use of medical correctives, as we already routinely disregard the consent of criminal offenders in incarcerating them. The onus is therefore placed on opponents to show why medical correctives require consent, when incarceration does not. Crucial to this approach being successful is that the permissibility of minimal incarceration is assumed for the sake of argument, due to its wide acceptability. However, while acceptability may provide motivation for the plausibility of minimal incarceration, this will not do in terms of grounding its justifiability. Although assuming certain premises is necessary for the sake of argument in philosophy, it seems problematic to presuppose as morally contentious a premise as the justifiability incarceration, particularly when further contentious implications such as the forcible imposition of medical correctives stem from its acceptance. In normative theory we are concerned with what we *ought* to do, and this is quite often detached from what we do happen to do. It may be mistaken, therefore, to view the issue of medical correctives through the lens of the Consent Requirement. Perhaps our practice of non-consensual incarceration should be critically re-appraised, rather than having its justifiability taken as an “accepted” foundational premise.

In order to undertake this appraisal, it is necessary to explore the reasons for the practice; the aims of punishment that its imposition is allegedly conducive to, and whether it can be seen as successful in these aims. As well as this, I consider the arguments put forward by Douglas that seek to demonstrate the parity by which medical correctives and incarceration inflict harm and threaten agency. Although his aim is to show that medical correctives are *no more harmful*, and *no more of a threat to agency* than incarceration, his analysis also sheds light on the severity of the harms imposed by incarceration. In light of such harms, his argument could plausibly lend support to the view that incarceration is indeed objectionable, and therefore may not do the work to show that non-consensual medical correctives are permissible, but rather, exposes the problematic nature of both.

Whether the Parity Claim can be used with success in Douglas’ argument, I argue, is dependent on whether it can be claimed that incarceration is a morally justifiable practice. This is because the argument that medical correctives are permissible insofar as they are sufficiently similar to incarceration, first assumes the permissibility of incarceration. Douglas acknowledges this assumption; “plausibly, in committing certain crimes, an offender becomes morally liable to the imposition of minimal incarceration, and for a substantial period. (In what follows, I will simply assume that this is so.)”^4^ The logic of the argument is as follows, therefore:

I. Non-consensual incarceration is a permissible practice.
II. There are various harms and threats to agency associated with incarceration.
III. Non-consensual medical correctives would inflict a comparable level of harm and pose an analogous agential threat.
IV. When two practices inflict a comparable level of harm and pose an analogous agential threat, they are to be judged as equal in terms of their moral import.

Conclusion

V. non-consensual medical correctives are permissible.

The argument appears to be that, in committing crimes and becoming “morally liable” to being incarcerated, criminals “might also become liable to the imposition of some varieties of medical intervention.”^5^ It is suggested, therefore, that “a proponent of the Consent Requirement owes us an explanation as to why medical interventions are *not* among the interventions to which we become liable.”^6^

Crucial to Douglas’ argument, and to my critique, is the fourth premise, which I have made explicit. For Douglas, there is no significant difference, morally speaking, between incarceration and a corrective injection. The *reductio ad absurdum* of this position, however, is that anything as harmful as incarceration is similarly permissible. This *reductio* is a direct consequence of the fourth premise, causing its soundness to be cast into doubt. It also results from Douglas’ dismantling of the Consent Requirement, and he offers us no principled way of deciding which punishments would be permissible, on his analysis, given we have already accepted consent is not required, and that rehabilitation, widely conceived, is the aim. This, I will argue, could plausibly force his account to concede that various other problematic practices, that he would not have wanted to include in his initial claim, are similarly permissible.

Perhaps anticipating an objection like this, Douglas is quick to point out that “it should not be assumed that, in challenging the Consent Requirement, I am defending the view that more invasive interventions—such as major surgical procedures—could permissibly be imposed without consent.”^7^ Yet, he provides no argument as to why certain other interventions would not be permissible, given the implications of his argument. Although he is clearly not arguing for this conclusion, he *is* challenging the Consent Requirement, and if successful, he would need to explain why other forms of punishment of the same intensity and level of invasiveness, which also aimed at “whatever higher goal rehabilitation serves”^8^ would not be permissible, as long as they were no more harmful than incarceration. The aim of the Parity Claim is exactly to show that the similarity between incarceration and medical correctives in various respects provides a challenge to the Consent Requirement; if it does not hold for one, why should it hold for another? This has important implications.

Rather than using a misleadingly extreme example like major surgical procedures, as Douglas does, in order to assure the reader that he could not be advocating for such a conclusion, let us instead consider a practice that would be sufficiently comparable to incarceration in terms of harm and threat to agency. Suppose that a new type of punishment was invented. Here, punishment is simulated as lasting longer, and a criminal’s own crime is simulated as being committed against them, in order to induce empathetic responses, which are said to facilitate rehabilitation. If it could be shown that any harm or distress caused by this was comparable to, and no worse than, the harms already inflicted by incarceration, then it seems that this would also be permissible on Douglas’ analysis. Further, it is not clear why this would be more threatening to agency than keeping someone under lock and key. This practice may even be more ‘efficient’ at rehabilitation, enabling the offender to be released sooner, and so could actually enhance their agency. Indeed, it is a struggle to think of anything that would be a greater threat to agency than the ultimate loss of liberty that incarceration entails, given that Douglas acknowledges the way in which incarceration can directly interfere with the mind.^9^ This is a potential problem for Douglas, as it means there are innumerable practices comparable to incarceration in this way, which could be levelled as instances of a *reductio*. The bar for comparisons of harm and threat to agency is therefore set very high, if incarceration is the benchmark.

Similarly, let us imagine a medical corrective is invented that regulates a released criminal’s sleeping patterns, causing them to sleep for 12 hours of the day. Consider it a medically-induced curfew. This is implemented with the view to reducing their involvement in the night-time economy, which for them, previously involved drugs, gangs, and violence. With the help of this drug, the criminal will now arise, like clockwork, at 6am, and experience 12 hours of the day, until around 5pm when they will start getting tired and prepare themselves for sleep, and eventually fall fast asleep at 6pm. This ensures they can no longer be led astray by the night-time culture that they once used to occupy. It seems that this would be a no greater threat to their agency than the practice of minimal incarceration, which already placed serious constraints on their freedom of movement. Indeed, they have greater liberty through this practice, as they are able to live in the outside world. In many ways, their agency would be greater enhanced through such a programme – the guarantee that they would be asleep by 6pm would enable them to make the most of their day, and the effectiveness of the procedure means they would not reoffend, and so would maintain their basic freedom by avoiding incarceration again. There is no reason to think this practice would be *any more* harmful than minimal incarceration, which already subjects prisoners to great harms regarding free movement and association, and in many ways, would perhaps even be *less* harmful, given they are removed from a potentially noxious prison environment. It seems that this is a practice that we would not be comfortable with implementing coercively however. Yet it fulfils the conditions of parity with incarceration, and would be thought of as ‘conducive to rehabilitation’ on Douglas’ analysis.

The position seems to be, therefore, that as we are permitted to harm criminals through incarceration, and if this is comparable to some other practice, then we are also allowed to harm criminals via this other practice, with the overarching reason being that their consent is already considered violable in the context of criminal justice. Yet this *reductio* makes the Parity argument appear unpalatable. It seems that the decision to deem a particular punishment permissible, is balanced against other considerations, not just its similarity with incarceration. I will return to this argument later. Douglas is clear to point out that his intention is only to propose that medical correctives *might* be justifiable, and that his argument “does not of itself establish as much,”^10^ as “there might be other moral reasons to prefer an approach in which medical correctives are offered as an optional alternative,”^11^ rather than being compulsory. But my argument is only to point out that it is not even clear that they *might* be justifiable on this reasoning, given the flaw in the soundness of this key *reductio*-inducing argument.

Further, in arguing that a parallel Consent Requirement does not hold for criminals, they are problematically differentiated as a *class* of people, to whom the Consent Requirement does not apply, categorically speaking. The reason that we incarcerate criminals, however, is not *because* we think it permissible to do anything to them without their consent, or because the Consent Requirement simply no longer holds for them in light of their offending. Rather, it is because of the particular *aim* of punishment that incarceration is intended to serve, be that rehabilitative, retributive or deterrent. This aim, whatever it may be, is what grounds the justifiability of incarceration. Consider; prisoners on America’s death row are not murdered *because*, or, *for the reason that*, their consent is violable. Rather, it is because of the intended *retributive* and *deterrent* effects (as unfounded as they may be) that this punishment is purportedly justified. That prisoners’ consent is violated in the process, is a corollary, or parallel effect, of the primary aims and justifications of the punishment. It is not the case that they become liable to punishment *because* of the violability of the Consent Requirement. Therefore, it does not seem enough to argue that the violability of their consent is what grounds *another* form of punishment.

It may be objected that it is surely a necessary component of a theory of punishment that the consent of offenders *is violable*, for their punishment to be permissibly meted out. This is why it seems justifiable to incarcerate offenders on rehabilitative grounds, and why it seems objectionable to incarcerate those who have not yet offended, but who may pose an equal risk of offending (given certain presumptions about their dispositions and background, say) also on rehabilitative grounds. The answer has to be that the offender has acted in a way that removes the Consent Requirement from applying, and therefore permits the forcible imposition of rehabilitative interventions. This may be true, but, as aforementioned, the fact that an offender’s consent is flouted is seemingly a consequence of the purported aims of punishment, not the main, motivating principle to be carried over elsewhere. Rather, the primary justification for incarceration is the positive argument for the purpose it is meant to serve, in this case rehabilitation, and the violability of an offenders’ consent is a consequence of, and is justifiable by, this purpose. It is not the case that offending leaves *any* non-consensual practice permissible – it is justifiable towards some end. The Consent Requirement no longer holds *because* of what we want to achieve in punishment, and it is not the case that violable consent *is* the justification for the punishment. Douglas, however, in focusing on the discrepancy in the application of the Consent Requirement, and in neglecting to outline the positive aspect of the argument for incarceration, is assuming that both incarceration and medical correctives can realise their rehabilitative goals, and so are justifiable. This is a step that I will turn to interrogate next.

Thus far I have attempted to show that the Parity argument is susceptible to an unappealing *reductio*, and that the violability of the Consent Requirement is only part of the story. It follows that the positive argument from rehabilitation for incarceration and medical correctives, is problematically lacking in Douglas’ account. Incarceration is justifiable with reference to, and from the standpoint of, a particularly theory of punishment. If it could be shown that the imposition of medical correctives was also justifiable under the aims and purposes of this same theory of punishment, then it would seem more plausible to use the permissibility of incarceration to justify the permissibility of medical correctives.

### Rehabilitation

This appears to be a missing step in Douglas’ argument – yes, it is widely thought it is permissible to do things to prisoners without their consent – but *why*? What is the purpose of this punishment that we impose non-consensually?

Douglas’ account assumes that both medical correctives and incarceration are justifiable by, and fulfil *rehabilitative* purposes. I suggest that both conceptually and empirically speaking, incarceration may not be able to deliver rehabilitation. Further, at the very least, I assert that it should not be safely assumed that it does, as Douglas’ argument demands. He briefly considers the objection that medical correctives and incarceration have different aims of punishment, and so cannot employ the same justification:

“Medical correctives are, we are assuming, employed in order to aid the offender’s rehabilitation. By contrast, incarceration, it might be argued, is intended to mete out deserved suffering, to communicate social disapproval, or to deter third parties from offending. Thus, one might argue that consent is required for medical correctives, but not for minimal incarceration, on the grounds that the goals of incarceration are different to, and perhaps morally more urgent than, the goals served by medical correctives. Perhaps it is permissible to nonconsensually treat offenders in intrusive ways in order to realise retributive, communicative or deterrent goals, but not in order to realise rehabilitative ones.”.^12^

He rebuts the critique that prison is justified on other grounds rather than rehabilitative, in claiming that rehabilitation “has commonly also been regarded as a goal, and in some cases, the only goal of incarceration,”^13^ citing the work of Alexis de Tocqueville and Gustave de Beaumont as a classic statement of the view that rehabilitation is the sole goal of incarceration. Further, Douglas argues that two other goals of incarceration, incapacitation and deterrence, are also “commonly thought to serve the same higher objective as rehabilitation: namely, the *prevention of crime* or, more generally, the *maintenance of security*.”^14^ This seems to problematically broaden the idea of ‘rehabilitation,’ however, and leads to the unpalatable conclusion that almost anything that fulfils these expansive goals can be done under the banner of rehabilitation, as long as it is not more harmful than minimal incarceration, leading to the kind of *reductio* discussed earlier. I will grant, however, that rehabilitation, however broadly conceived, *is* ostensibly considered to be the purpose of incarceration, as opposed to say, retribution. I will now turn to explore whether incarceration is successful in this purpose, and so whether it may plausibly be justified this way.

### The Problem of Prison

There appears to be potential difficulty in appealing to rehabilitative aims to justify the practice of incarceration. Even if we accept Douglas’ broad definition of rehabilitation as “crime prevention,” it is questionable as to whether incarceration is indeed conducive to this aim.

Douglas is correctly quick to point out that our practice of incarceration as it currently stands is:

“hard to justify given prevailing prison conditions, which often involve exposing incarcerated individuals to overcrowded conditions, a high risk of rape and assault, and serous health threats.”^15^

Instead, he asks us to imagine a utopian prison system that:

“placed serious and constant constraints on free movement and association, but otherwise exposed offenders to no greater risks to their health and security than average members of the unincarcerated citizenry, and took all reasonable steps to safeguard opportunities for political participation, legal representation and education.”^16^

Imagining a tweaked prison system that would be less damaging to criminals’ physical wellbeing, and with purportedly improved rehabilitative functions, Douglas does not broach the question as to whether incarceration actually *is* the most effective method of crime prevention. Yet, it seems that the issue remains as to whether a punishment which still placed such “serious and constant constraints” on free movement could properly be conceived as rehabilitative, as compared with say, a non-custodial sentence. Under a minimal incarceration model, offenders would still be exposed to psychological and other health risks, through constraints on their movement and association, in a way that might impact upon their rehabilitation. Indeed, it is being assumed that any risks imposed that are a consequence of these “serious and constant” constraints and curtailments, are permissible and not antithetical to rehabilitation.

Yet it is widely accepted that prison has an adverse effect on an already vulnerable population’s mental health. A recent report from the All-Party Parliamentary Group (APPG) on Prison Health suggested that prisons continue to be used as institutions for individuals with mental health problems, and recommended that other institutions might cater for their needs more effectively. They cited the Office for National Statistics (ONS) survey of mental ill heath in the prison populations in England and Wales in 1997 which indicated that “90% of prisoners have at least one mental health disorder, including personality disorder, psychosis, neurosis, alcohol misuse and drug dependence [1].”^17^ The majority of that number have ‘common’ mental health problems such as depression and anxiety, and it was suggested that much of this is likely to be related to their imprisonment rather than having been a contributing factor to it, indicating that incarceration has deleterious effects on mental health.

This cuts to the heart of the debate: can prisons, which unavoidably affect individuals in this way, truly serve their rehabilitative function? Even the most idealised prison system, that wasn’t a ‘school for crime,’ had various educational programmes in place, and took steps to ensure a job on release, would plausibly have an effect on an individual’s psyche. Nevertheless, if such a utopian prison system *were* successful in its aims, it would seem that this success would be down to more than the feature of ‘locking people up,’ qua incarceration. It would be all the other aspects; the vocational programmes and efforts to reintegrate prisoners back into society, that fulfil punishment’s rehabilitative aims, rather than the act of incarceration itself. Indeed, such initiatives plausibly do much to combat against the negative psychological and social effects associated with incarceration. This raises the question as to whether the custodial part of the sentence – incarceration itself, is indeed a necessary or morally justifiable practice to impose upon offenders, if the rehabilitative function could be served through other means. There is reason to suppose that in ideal circumstances, a more just punishment system would incarcerate only the most dangerous criminals, that are beyond psychological help. One could also point to the Scandinavian model as evidence that lower incarceration rates and shorter sentences are more conducive to low recidivism rates [2, 3],^18^ a pattern that points to the counterproductive nature of imprisonment, even the minimal incarceration model that Douglas suggests.

Separate to the social scientific arguments surrounding crime prevention, there is a rich debate amongst philosophers concerning whether *punishment* is a morally justifiable practice per se. Douglas’ argument rests on our acceptance of the assumption that the practice of minimal incarceration is permissible even in his imagined situation. As aforementioned, he notes that it would be “widely (though not universally) accepted that the state could permissibly impose conditions of this sort…on at least some criminal offenders.”^19^It is instructive to note that he uses the language of ‘accepted’ – such a practice would be *accepted*, rather than *justifiable*. This is seen as sufficient motivation to ask us to accept it for the sake of argument. Suffice it to say that many practices would be, and are, widely accepted, despite not being morally justifiable. Legal tax avoidance and the death penalty in the USA serve as two such examples. Yet it seems methodologically problematic to argue for further, similar practices based on their similarity to an already accepted, but admittedly controversial, practice. That we may justifiably non-consensually incarcerate criminals is taken as a given by Douglas, when in fact this is a contentious point of debate. Perhaps most famously, Ted Honderich has condemned the practice of incarceration in his book *Punishment*: *The Supposed Justifications*. He argues that of the three main theories of punishment, deterrence, retribution, and rehabilitation, none can sufficiently justify the practice [4].^20^ Although there is not enough space here to outline his case, I believe he provides a compelling, and at the very least, *plausible* account of why even Douglas’ minimal incarceration model would be unjustifiable. Though there is little doubt that Douglas’ view would be widely *accepted*. Deidre Golash similarly argues that utilitarian and retributive justifications for punishment fail, both conceptually and empirically [5],^21^ while David Boonin argues that purported solutions to the problem of punishment – why it is permissible for the state to treat those who break the law differently from those who do not – are unsatisfactory, proposing victim restitution as an alternative to punishment [6].^22^ Elizabeth Anderson considers mass incarceration to be “modern outlawry,” removing ordinary protections of citizenship from already stigmatised groups. Far from deterring crime, she argues, (and conceivably, from aiding rehabilitation, I add), this practice invites the criminal victimization of outlaw groups, undermining the rule of law, equality under the law, and a democratic culture [7].^23^

Increasingly theorists are submitting that our current practice of punishment is failing in its aims, and therefore cannot be justified by those same aims. As well as this, it is argued that punishment is problematic per se, not just our current, flawed approach, casting doubt on the justifiability of Douglas’ alternative minimal incarceration model. Yet, it remains an *accepted* practice, in terms of policy. Douglas may be reasoning from the socially and historically contingent fact that we currently *do* incarcerate, but this does not mean that we *ought* to. It may be problematic to use this practice as a springboard for justifying further similar practices, therefore. Although this paper has used the words ‘permissibly’ and ‘justifiably’ somewhat interchangeably, I am concerned primarily with justifiability, as I consider this prior to permissibility. From the point of view of ideal theory, therefore, it seems plausible that we would not want normative, guiding principles to be shaped and constrained by current dogmas, particularly if there are reasons to doubt such beliefs.

### Incarceration and Harm

Thus far it has been suggested that incarceration may not be justifiable on rehabilitative grounds. It could plausibly be maintained that the reason there is a discrepancy in Douglas’ imagined interlocutor’s thinking – that the Consent Requirement does not hold for incarceration, but does for medical correctives – is because neither are truly justifiable, and so ideally, neither ought to be non-consensually imposed. The imagined interlocutor might maintain that incarceration is indeed harmful, but that a line must be drawn somewhere, in regards to what further harms we may permissibly impose upon prisoners, even though we already permissibly expose them to great harms. On such an approach, Douglas’ arguments to show that medical interventions are only as harmful or problematic as minimal incarceration, could actually serve as arguments *against* minimal incarceration, and as such, are not sufficient in establishing the permissibility of forced medical correctives. In acknowledging how much harm incarceration already inflicts, Douglas’ analysis could lead one to question whether we should indeed be imposing this practice at all, if we recognise it as harmful.

Douglas considers the idea that the right to bodily integrity could be invoked in defence of why the Consent Requirement holds for medical correctives, but not incarceration, and therefore why the former are thought to be impermissible. An imagined interlocutor maintains that the specific rights to bodily integrity that protect against injection, as in a medical corrective, are *more robust* than the specific rights to free movement and association that are violated under minimal incarceration. The exponent of such a view is making the Robustness Claim:

“It takes more serious criminal offending for the rights to bodily integrity that protect against injection to lose their protective force than for the rights to free movement and association that protect against minimal incarceration to lose theirs.”

Such an individual holds that the Consent Requirement may be flouted for incarceration, therefore, but not for medical correctives, given the robustness of the right to bodily integrity. What is the basis for such a claim? Douglas considers the argument that the right to bodily integrity is more robust than others because it protects against forms of treatment that typically cause serious harm, more serious than constraints on free movement and association involved in minimal incarceration. He responds that it is difficult to see how this consideration could be invoked to defend the Robustness Claim, because the restrictions on movement and association entailed by incarceration would *also* “reliably cause (and may themselves constitute) significant harms.”^24^ This is because incarceration and the restrictions it entails “would frequently damage existing personal relationships while making it difficult to form new ones, they would seriously restrict sexual freedoms, they would make it impossible to pursue most careers, and they would more generally prevent the realisation of many life-plans,” and at the very least, would “cause significant distress.”^25^ Douglas admits, therefore, that minimal incarceration is “severely harmful,” in most cases. But this point is not intended by him to show that incarceration is morally problematic, but rather, that “it is difficult to see why we should expect that the imposition of an injection would normally inflict *more* harm.”^26^ Yet it seems plausible to suggest that exposing prisoners to this level of harm, as Douglas admits we do, might hinder their rehabilitation in some way, furthering the argument that it is unjustifiable on such grounds.

Douglas similarly dismisses the argument that the robustness claim is to be defended via an appeal to the protection of agency. He argues there is no reason to think “that interfering with someone’s body by injecting a drug (typically) constitutes a more grave threat to agency than does constraining his freedom of movement and association in the way entailed by minimal incarceration.”^27^ Further, he argues that it is not the case that interfering with the mind, as would occur with medical correctives, constitutes a more severe threat to agency, than merely removing options, as in minimal incarceration. He suggests that such a view might rest on an assumption that interfering with the mind is more threatening to agency due to the way in which it interferes with agency at its *roots*. However, he makes the insightful point that constraints on free movement can *also* interfere with the mind, and so agency at its roots. This is because the mind is “dependent on, and influenced by, our immediate natural and social environment, which in turn is affected by restrictions on free movement and association.”^28^ On such a view, minimal incarceration can also directly influence the mind. These arguments present non-consensual minimal incarceration as incredibly invasive and harmful. In another context, they could plausibly be used to provide a strong case for the reassessment of the practice. They do not appear to necessarily do the work to show that another analogously harmful practice should also be permissible, but rather, shed light on the problematic nature of both.

### Medical Correctives and Rehabilitation

In light of this, a proponent of medical correctives might make the alternative case that, in bypassing much of the harms associated with incarceration, such correctives are a more appealing practice. If we could aid prisoner’s rehabilitation with the use of a brief injection, without subjecting them to the harms caused by incarceration, then perhaps this would be preferable. This argument would require a prior justification for medical correctives on rehabilitative grounds, however, as the purported justifiability of incarceration is no longer being used as the grounding. Indeed, correctives are being presented as an *alternative*, given the problems associated with incarceration. Can they be justified using a standalone theory of rehabilitative punishment, therefore?

That medical correctives can in fact aid rehabilitation is something that Douglas takes as a given, without argument. Yet the question as to whether medical correctives are conducive to the aims of the punishment according to which their implementation is justifiable, should be explored. How might this justification go, therefore? Douglas states that research is said to have uncovered the “neural correlations of dispositions towards aggression, impulsiveness, diminished empathetic ability and psychopathy.”^29^ He is implicitly associating these traits with criminal activity, a connection which requires further justificatory support. Correspondingly, scientists are also “suggesting means of influencing these dispositions in ways that might be thought conducive to rehabilitation in some offenders.”^30^ Douglas does not opine exactly how altering such dispositions would constitute rehabilitation or why altering these traits would yield the desired results, nor does he outline which sorts of offenders would be targeted, or for which crimes. Yet these are all key steps to explaining how medical correctives would fulfil a rehabilitative function. He provides the examples that anti-depressants have “shown promise in reducing aggression,”^31^ and that “divalproex has been found to reduce impulsiveness in adolescents with explosive temper.”^32^ It appears that the direction of current research leads him to conclude that “we will have available a significant range of medical interventions capable of aiding rehabilitation.” However, I believe there are a number of assumptions in this view that require unpacking. It is no good to assume for the sake of argument that such drugs will aid rehabilitation. Whether such drugs really *can* facilitate rehabilitation is inextricably and interdependently tied up with their permissibility. Indeed, whether such drugs would actually aid rehabilitation, and so be permissible, depends on how rehabilitation itself is defined, and what it is theorised to consist in.

The following set of premises appear to be foundational to Douglas’ argument:

1. there are neural correlates of dispositions towards aggression, impulsiveness, diminished empathetic ability and psychopathy
2. the discovery of these neural correlates allows us to plausibly identify and alter the dispositions they correlate with
3. such dispositions cause people to commit crimes, or at the very least, appear to have a significant impact on whether or not people commit crimes
4. altering these dispositions can constitute rehabilitation in some criminals, as they would no longer be inclined to commit crime

These premises appear to indicate that criminal activity has mental causes that are, to some extent, determined. Douglas may interject that he is no where claiming that crime is *fully* determined by such dispositions, and that his account is consistent with an interactionist model of crime, that holds that these dispositions interact with certain environmental circumstances to cause an individual to commit a crime. However, this is not what his account *does* state, and given his approach neglects any such mention of the social causes of crime, it is worth noting the ways in which a more holistic social view might present a challenge the approach he invokes, and render it incomplete. It may plausibly be suggested that dispositions do not vary that dramatically between individuals. Dispositions could be conceived of merely *as* dispositions, that, until triggered, lay dormant. What *does* vary more dramatically between individuals, however, and *does* affect whether such dispositions come into play, is the environment, and the conditions that facilitate their manifestation. The kind of approach that views the individual as the appropriate focus of intervention to control dispositional risk factors of crime, therefore, fails to address exactly *why* some people go on to commit crime, with such dispositions. It is in this sense that the social conditions of crime are neglected from such a narrative.

This approach also appears to ‘other’ criminals in its assumption that they are morally bankrupt, or at least, somewhat different in character from the rest of the population. The language used in the paper, such as the intention that the “post-rehabilitation offender will be a *morally better person*,” conflates morality with criminality, and posits criminals as morally deficient, as a general class, in virtue of their offending. This cannot be the case, as the supposed immorality of an action does not seem to be either necessary or sufficient for its being illegal. For instance, whilst most would agree that extramarital affairs are morally wrong, they are not a crime. Conversely, while recreational drug use *is* illegal, it is not clear how exactly this behaviour is ‘immoral’ (when the issue of mere use is considered separately from problems such as drug-related violence). It may be the case that committing crimes with disregard for the social consequences does comprise flawed behaviour, but it may also be inaccurate and counterproductive to label criminals in such a divisive way. It seems equally plausible that criminals deemed ‘immoral’ by the state could exhibit moral traits such as empathy and have ‘moral motives’ in their own personal relationships. The language of ‘morality,’ in discussions on the treatment of offenders, therefore, should be viewed with caution. The idea of the state prescribing ‘morality’ in this way is reminiscent of the forced use of hormonal treatments to reduce libido, when homosexuality was criminalised and considered ‘deviant’ behaviour. Such a practice is now condemned because we acknowledge that homosexuality should not have been considered immoral, or a crime. While offenders in this case *have* committed a crime, the point remains that this does not necessarily indicate a flaw in their morality, and so attempts to ‘treat’ this as such could be misguided. Further, without further explication as to how certain crimes do indicate a flaw in an individual’s morality, and how medical correctives would eradicate this flaw, the discussion seems to problematically presuppose too much.

The idea that criminals as a class are to be distinguished in terms of their dispositions towards crime is reminiscent of the positivist school of criminology of the late nineteenth century, which attempted to find psychological and biological bases for criminal behaviour. The dominance of positivist criminology declined after the 1960s when sociological criminology came into the fore, which led to a shift towards seeing crime as the outcome of social circumstances [8]. In particular, the Chicago School provided a strong challenge to the individualistic focus in Britain, and, for the first time, treated the “city”, or environment, as an issue. This approach was characterised by relating delinquency “to the nature of the social processes associated with the areas in which it occurred.” Similarly, the American sociologist, Robert Merton, located the source of criminal behaviour in relative deprivation, and considered phenomena such as property crime as structural issues, and the result of a lack of equality of opportunity. This cursory discussion of different criminological approaches is intended to show that there exists a rich and varied debate regarding the causes of crime that Douglas fails to acknowledge in assuming that biological dispositions can have enough bearing to merit intervention at that level. Positivism operates under the guise of scientific objectivity, but is itself an ideological position, positing the individual as the ultimate unit of concern, to the neglect of social determinants. In so far as his argument for medical correctives implicitly operates on this model, it is equally liable to the challenges posed to positivism by competing theories of crime. Rather than taking positivism as an axiomatic premise, therefore, proponents of medical correctives ought to make explicit, and subsequently defend, the use of positivism in their argument. Even if Douglas maintains that his argument for medical correctives is intended to compliment, rather than *replace* a concentrated effort in addressing the social determinants of crime, the connection between crime and the rehabilitative success of the proposed punishment remains to be shown. It is not explicitly defended, but rather, is assumed, which proves problematic if a standalone case for the rehabilitative purpose of medical correctives is what is required.

Further, there appears to be no scientific consensus on the issue as to whether crime *can* be treated at the dispositional level. These practical considerations are important because they may betray a flaw in the understanding of the causes of crime, and so risk offering a mistaken solution. This problem is highlighted in an examination of the use of chemical castration on convicted sex offenders. Chemical castration via administration of the testosterone-lowering drugs has been found effective in reducing recidivism in sexual offenders with paraphilias in some small-scale, controlled studies, while others have found no significant effect, indicating that evidence for effectiveness is not robust [9]. Even in studies which do appear to show lower recidivism rates in those who have undertaken surgical castration, compared with those who have not, John McMillan warns that we should be cautious about the quality of the controls. This is because the group who did not undertake the procedure, and had higher recidivism rates, had been counselled about the possibility of castration but had decided against the operation. McMillan posits that it might be the case that “their decision to not follow through with operation was because of a lack of resolve to control their offending, whereas men who were prepared to have this operation might have been more willing to do whatever it takes to change their behaviour [10].” The low recidivism rate, therefore, may have been attributable to their will and desire to change, as indicated by their compliance with the procedure, or at the very least, it remains *unclear* what their success is a result of. Assuming these practices work even ‘for the sake of argument,’ therefore, is to make a rather large assumption about the causes of crime, an area of perennial debate.

### Social Considerations

Suppose a clear link between the imposition of medical correctives and rehabilitation had been established. This link was enough to show that they could be justified as a form of punishment, under this purpose. Is this enough to show that they may permissibly be imposed, however? I do not believe it is. Crucially, just because a punishment can be justified under the aims of a particularly theory, this does not do enough to show that it is permissible, or that it *ought to be implemented*. Douglas admits that his argument does not do enough to show the justifiability of medical correctives, merely the *possibility* that they are justifiable. I believe it is still worth noting, however, the problems associated with suggesting it as a possibility, given other considerations.

While crime prevention is the primary purpose of our criminal justice system, and a given punishment may be thought conducive to its fulfilment, this purpose does not exist in a vacuum. It is neither the only, nor necessarily the most important social purpose. There exist other social purposes that must be taken as balancing considerations. These include, say, the fulfilment of justice, particularly relational, the enhancement of democratic freedom, and the attainment of social cohesion. Such purposes may plausibly be undermined by the further stigmatisation and categorisation of prisoners, which could occur through the use of medical correctives. Although partly an empirical question, it would not be unreasonable to suggest that such treatments could have adverse effects, given the existing demonisation of, and stigma faced by ex-convicts. Such parallel social purposes, therefore, could conceivably serve to qualify and place limits on the goal of crime prevention, deeming medical correctives impermissible per se. Even if the Parity Claim were based on more sound reasoning concerning the justifiability of incarceration and the equal moral import of incarceration and medical correctives, Douglas would still face a key hurdle. The requirement that various social purposes need to be balanced against each other, shows that the analogy cannot be stretched to an extent that allows us to conclude that the infliction of a comparable harm is even ‘theoretically’ permissible in this case.

### Conclusion

The aim of this paper has been to cast doubt on the viability of Douglas’ parity approach in reasoning about the permissibility of non-consensually imposing medical correctives on criminals. It has been argued that prisoners’ consent being violable in the context of incarceration is not sufficient reason to ground the imposition of other punishments non-consensually. Indeed, removing the requirement for consent in this way leads to an unpalatable *reductio ad absurdum*, whereby any practice just as harmful as incarceration seems to be permissible. Rather, the justifiability of a particular punishment is grounded in its aim towards fulfilling a particular purpose. Therefore, for the parity of reasoning approach to carry any weight, and for the structure of the argument to be applicable, medical correctives and incarceration need to be shown as aiming towards the same purpose, which, in this case, is taken to be rehabilitation. It has been suggested, however, that incarceration may not be justifiable under rehabilitative aims, and therefore may not provide the grounding for the justifiability of medical correctives, that is required of it. That incarceration is justifiable according to rehabilitative reasoning is at least plausibly disputable, given both empirical and conceptual concerns about the practice. Further, it has been suggested that an independent case that medical correctives are justifiable according to rehabilitative purposes, may also be difficult to make. There are various assumptions contained within the view that they can aid rehabilitation, particularly the very basic idea that they *will* do so at all, given that rehabilitation is a perennially debated area of criminology. Finally, even if the case could be conclusively made that medical correctives were conducive to rehabilitation, it is unlikely that it could be shown that they may permissibly be imposed. While crime prevention is a key social purpose of the state, it seems that other social purposes serve to constrain and limit the practices which may be imposed under this aim.

## Footnotes

1 Ibid., p. 105

2 Ibid., p. 104

3 Ibid., p. 105

4 Ibid.,

5 Ibid.

6 Ibid. (emphasis added)

7 Ibid., p. 104

8 Ibid., p. 106

9 Ibid., p. 117

10 Ibid., p. 120

11 Ibid.

12 Ibid., p. 106

13 Ibid.

14 Ibid. (emphasis added)

15 Ibid., p. 105

16 Ibid.

17 Douglas, “Moral Liability,” p. 105

18 Douglas, “Moral Liability,” p. 114

19 Ibid.

20 Ibid.

21 Ibid., p. 117

22 Ibid.

23 Ibid., p. 103

24 Ibid. p. 102

25 Ibid.

26 Ibid.

27 Ibid.

28 Ibid.

29 Ibid.

30 Ibid., p. 101, emphasis added

31 Ibid., p. 84

32 Ibid.
